# Human *Trypanosoma cruzi* chronic infection leads to individual level steady-state parasitemia: Implications for drug-trial optimization in Chagas disease

**DOI:** 10.1371/journal.pntd.0010828

**Published:** 2022-11-21

**Authors:** Pablo M. De Salazar, Sergio Sosa-Estani, Fernando Salvador, Elena Sulleiro, Adrián Sánchez-Montalvá, Isabela Ribeiro, Israel Molina, Caroline O. Buckee

**Affiliations:** 1 Center for Communicable Disease Dynamics, Department of Epidemiology, Harvard T.H. Chan School of Public Health, Boston, Massachusetts, United States of America; 2 Drugs for Neglected Diseases *Initiative*. Rio de Janeiro, Brazil; 3 Epidemiology and Public Health Research Centre, CONICET, Buenos Aires, Argentina; 4 Department of Infectious Diseases, Vall d’Hebron University Hospital, International Health Program of the Catalan Institute of Health (PROSICS), Barcelona, Spain; 5 Department of Microbiology, Vall d’Hebron University Hospital, International Health Program of the Catalan Institute of Health (PROSICS), Barcelona, Spain; 6 Drugs for Neglected Diseases *Initiative*, Geneve, Switzerland; 7 Instituto René Rachou-FIOCRUZ Minas, Laboratório de Triatomíneos e Epidemiologia da Doença de Chagas, Belo Horizonte, Brazil; 8 Centro de Investigación Biomédica en Red de Enfermedades Infecciosas (CIBERINFEC), Instituto de Salud Carlos III, Madrid, Spain; University of Texas at El Paso, UNITED STATES

## Abstract

Currently available drugs against *Trypanosoma cruzi* infection, which causes 12000 deaths annually, have limitations in their efficacy, safety and tolerability. The evaluation of therapeutic responses to available and new compounds is based on parasite detection in the bloodstream but remains challenging because a substantial proportion of infected individuals have undetectable parasitemia even when using diagnostic tools with the highest accuracy. We characterize parasite dynamics which might impact drug efficacy assessments in chronic Chagas by analyzing pre- and post-treatment quantitative-PCR data obtained from blood samples collected regularly over a year. We show that parasitemia remains at a steady-state independently of the diagnostic sensitivity. This steady-state can be probabilistically quantified and robustly predicted at an individual level. Furthermore, individuals can be assigned to categories with distinct parasitological status, allowing a more detailed evaluation of the efficacy outcomes and adjustment for potential biases. Our analysis improves understanding of parasite dynamics and provides a novel background for optimizing future drug efficacy trials in Chagas disease.

**Trial Registration:** original trial registered with ClinicalTrials.gov, number NCT01489228.

## Author summary

Better understanding of the *Trypanosma cruzi* parasite dynamics in the bloodstream of chronically infected individuals is critical to improving existing treatments against Chagas disease, as well as to support the development of new therapeutics. Here, we characterize the dynamics of parasitemia over time in two cohorts of individuals suffering from chronic Chagas disease based on quantitative molecular methods and using statistical modeling. We found evidence that parasitemia remains at a steady-state in untreated individuals for long periods of time. The steady-state can be quantified and predicted at an individual level. This novel analysis framework in chronically infected individuals allows improving the evaluation of outcomes in clinical trials, and provides a new background to further understanding the clinical impact of *T*. *cruzi* infection.

## Introduction

Chagas disease is a neglected parasitic disease associated with poverty, which is endemic in North, Central and South America [1,2]. It is caused by *Trypanosoma cruzi*, and currently affects over 7 million people worldwide. It can lead to severe chronic morbidity, such as cardiomyopathy and/or severe digestive damage, which in turn can be fatal causing more than 12000 attributable deaths per year [1]. In the last two decades it has also become an important public health challenge worldwide due to population flows and persisting poverty [3,4]. While transmission mostly occurs through the bite of an infected vector, oral, mother-to-child, and blood borne transmission routes contribute to the global disease burden [1]. Beyond implementing public health interventions for early diagnosis and treatment, one of the largest obstacles to tackling Chagas disease is the lack of an early marker for monitoring therapeutic response, particularly for adult patients. Furthermore, curative treatment is based in two older compounds which, although they are efficacious in the acute stage, are difficult to evaluate for chronically infected adults, with efficacy estimates ranging between 2% and 40% based on serological or parasitological assessments [5,6]: treatment regimens are often long, and have a poor safety profile, leading to treatment discontinuation in a proportion of patients. The lack of early markers of cure has hindered a proper assessment of existing or repurposed drugs and the development of new compounds [7].

Serological testing remains the gold standard for diagnosis and subsequent monitoring of treatment success. However, it can take from years to decades after an individual is cured for levels of antibodies against *T*.*cruzi* to reduce significantly [8]. In recent years, clinical trials have used *T*. *cruzi* DNA detected by polymerase chain reaction (PCR) in peripheral blood as the main efficacy endpoint (i.e., sustained negative PCR at an arbitrary number of timepoints during follow-up after the end of treatment) [9,10]. However, while the interpretation of a positive PCR test after a full treatment course represents treatment failure, not detecting parasite DNA in blood samples is not accepted as demonstrating cure. For example, performing PCR on the blood of chronically infected patients known to be infected at a single time point results in a high proportion of non-detectable parasitemia (20–60%), aligned with findings using alternative methods (i.e., microscopy, hemoculture and xenodiagnosis [11]). High rates of non-detectable parasitemia are generally attributed to a stochastic intermittent absence of parasitic DNA due to the parasite life cycle in the host, although the accuracy of the diagnostic methods, particularly those with low sensitivity can also influence the detection rates [12]. Great efforts have been made to increase the diagnostic sensitivity over the last decade [13], including their use in clinical trials to assess new treatments [14] but a high proportion of patients still have negative results when PCR is performed from on *ex vivo* samples [11]. Systematic approaches assessing Chagas parasitemia within both quantitative and longitudinal frameworks are lacking, as most of the literature describes cross-sectional and/or qualitative interpretations [15–19].

Only individuals with detectable parasite DNA detection at screening are included in clinical trials, meaning that efficacy trials might be biased because they only assess treatment response in subjects with detectable parasitemia; this might be important if bloodstream parasite load is representative of the overall infection burden and subsequent response to treatment [14]. Thus, the current use and interpretation of PCR involves a high degree of uncertainty and cannot be relied on to predict an individual’s parasitological burden.

Here, we analyze multiple serial data sets obtained using quantitative (q) PCR against parasite DNA collected regularly over a year from untreated individuals with chronic infection, which allows individual-level quantitative characterization of the temporal dynamics of *T*.*cruzi* in the bloodstream. We show that parasite DNA detected by qPCR fluctuates around an individual-dependent steady-state during one year of follow-up. Applying this approach to individuals pre- and post-treatment can substantially reduce the uncertainty and potential confounders when evaluating therapeutic responses in efficacy trials, compared to the qualitative interpretations currently used as primary endpoints.

## Materials and methods

### Ethics statement

For individuals included in dataset 1, ethical approval for this research was granted by the Vall d’Hebron University Hospital Institutional Review Board. For individuals included in datasets 2 and 3 (original trial registered with ClinicalTrials.gov, number NCT01489228), ethical approval was granted by the Ethical Review Boards of the Universidad Mayor de San Simón, the Fundación CEADES, the Hospital Clínic, and Médecins Sans Frontières. Written informed consent forms were signed by all volunteers of both studies and possible consequences of the studies were explained (no minor subjects were included). All samples were anonymized before being processed.

### Datasets

For our analysis, we used 3 datasets. Dataset 1 (D1) comprised quantitative (q) PCRs performed regularly every 2–3 months from samples of non-treated individuals diagnosed with chronic Chagas disease by serology (n = 38) at the Vall d’Hebron University Hospital (Spain) and subsequently monitored for disease development from 2011 to 2013 when benznidazole was in short supply [20] Quantitative PCR was performed twice from every (single) sample per visit (i.e., timepoint). Most individuals in D1 (37 out of 38) were originally from the Bolivian departments of Cochabamba (n = 18), Santa Cruz (n = 14) and Chuquisaca (n = 5), but remained in Spain during the follow-up, and thus were not at risk of reinfection. All individuals had either chronic indeterminate status (n = 31) or with digestive damage (dolichocolon n = 5, megacolon n = 2), and none with cardiological involvement. Once the drug was available, patients were treated according to national guidelines. Dataset 2 (D2) comprised qPCR results from individuals included in the placebo arm (n = 47) of a randomized clinical trial evaluating a new candidate compound (E1224) against chronic Chagas [10]; and dataset 3 (D3) comprised individuals included in the four treatment arms of the same study: benznidazole (BDZ) at 5 mg/kg daily, 60 days, n = 45; candidate E1224 at a high dose (HD) 400 mg/kg weekly with an starting load of 400mg/kg daily on days 1–3, for a total of eight weeks (8w), n = 45; candidate E1224 at similar HD,for a total of four weeks, n = 46; or candidate E1224 at a low dose (LD) 200mg/kg weekly with a starting load of 200 mg/kg on days 1–3, for a total of 8w, n = 48. The study population of D2-D3 had been recruited and followed up in two outpatient clinics in Bolivia (Departments of Cochabamba and Tarija), after confirmation of a positive PCR at screening. Patients were then followed up for a year and qPCR was performed at baseline (before treatment), and at 10 weeks, and 4, 6 and 12 months after treatment. For this dataset, qPCR was performed 3 times in 3 different samples per each time point, 2 on the same day and one 7 days later [14]. A patient was considered PCR positive if at least one qPCR replicate showed detectable parasitemia. In both datasets, the median cycle threshold (Ct) value of all measurements below 40 (the cut-off value for detection in both dataset procedures) was used for each time point (up to 2 qPCR replicates in D1, and up to 9 replicates in D2-D3). Further details are included in S1 Text.

### Qualitative assessment of parasitological status in chronic Chagas disease patients over time

First, we aimed to explore parasite positivity dynamics using longitudinal qualitative PCR results in non-treated individuals included in D1 and D2. A “positive” result represents detection of parasite DNA in the bloodstream while a “negative” result represents non-detection (i.e., Ct-value over the cut-off threshold of 40). In particular, we aimed to test whether changes in parasitemia would be consistent with a binomial distribution, under the assumption that the probability of having detectable parasitemia is independent of time point. This null model represents a system where detection depends on the suboptimal accuracy of the diagnostic test. As the simplest alternative model, we hypothesized that individuals could be grouped in two categories, each category having an independent probability of parasite detection. In this alternative model, the individual probability of detection is interpreted as being conditional on being in one category, and the probability depends upon the accuracy of the diagnostic test for that category.

Thus, to evaluate the parasite bloodstream dynamics in untreated individuals, we formally approach the analysis for the not-treated individual time series included in D1 and D2 (individuals included in the placebo arm of the clinical trial) with at least one positive observation and at least 4 consecutive assessments (n = 26, and n = 46 respectively) using a hidden Markov model (HMM) [21]. We performed the analysis separately for each of the datasets. Briefly, a HMM represents the system as a Markov process, a stochastic model describing a sequence of possible events in which the probability of each event depends on the state attained in the previous event. The HMM includes “unobserved”, hidden states (herein categories). For our analysis, the null hypothesis is represented as a single category with a Bernoulli distribution for the event of detection. The simplest, alternative hypothesis includes 2 categories, each presenting a different probability of parasite detection, again represented by Bernoulli distributions, each with independent parameterization. The schematic model representations of the system are shown in Fig A in S2 Text. The HMM framework allows the estimation of the transition probabilities between categories (i.e., for each time step, what is the probability of moving towards another state?) as well as the emission probabilities conditional to each category (i.e., what is the probability for being positive given the current category?), using the Expectation-Maximization algorithm, which provides an iterative solution to maximum likelihood estimation with latent variables [21], and can handle missing observations obtained at regular time points under the missing-at-random assumption by maximizing the complete dataset log likelihood and directly estimating the parameters [22]. We estimate the maximum joint log-likelihood for all individuals with at least one positive result per dataset. Model selection is performed using the Akaike (AIC) and the Bayesian (BIC) information criteria [23]. Using HMM instead of a general mixture model allows the estimation of the individual probability of changing between categories at regular time points, which can be used to interpret the sensitivity of the model. Statistical details of the model can be found in S2 Text. The model was implemented in R using the *LMest* package [24].

### Quantitative assessment of parasitological status in chronic Chagas disease patients over time

Second, we aimed to evaluate the quantitative values of the PCRs performed over time, reported as Ct-values. For all datasets, the limit of detection was set at 40 Ct; thus, parasitemia leading to values over 40 were truncated. Of note, Ct-values approximate an exponential representation of the parasitemia (in an inverted scale) depending on limitations such as the number of copies of the targeted site per parasite [25] and multi-clonality [26,27].

We evaluated the individual qPCR trajectories in D1 and D2 among those presenting at least one negative measurement (i.e., Ct = 40) vs. those with only positive results (i.e., all Ct-values < 40). For each individual’s series of Ct-values we assumed that the median approximated the steady-state; we then assessed the distribution of the relative difference between each individual’s time point Ct-value and their steady-state as the relative approximation error. Furthermore, for each category defined in the previous step in both datasets, we computed the mean absolute percent error (MAPE). Lastly, to evaluate how adding observations improves the relative approximation error, we computed the MAPE using the average of two and three Ct-values at consecutive time points for each individual.

Using Ct-values instead of interpolating parasite load based on an experimental standard curve, which typically requires assuming a linear relation between the logarithm of the parasite concentration and the Ct-value, facilitates statistical analysis in a scale with biological interpretation, where 10-fold change in parasite load corresponds roughly with a change of 3.3 Ct [25], while does not require further assumptions for values under the limit of quantification, a common problem with clinical samples in the lower range of parasite load [28].Thus, here we assumed that median Ct-values from 2 replicates (D1) or up to 9 replicates (D2-D3) reasonably approximate the parasite load in the bloodstream at the time of the assessment. Direct comparison of Ct-values among individuals with different infections due to clones from different DTUs can be missespecified, as they might correspond to different parasite load because of differences in the number of targeted copies (here, satellite DNA [25]). However, given that individuals from D1 and D2-D3 were infected in Bolivia, and the majority coming from the same region (Cochabamba and Santa Cruz in D1 and Cochabamba and Tarija in D2-D3), and that we focus on individual-level trends, the approach is tenable for comparing individual trends within each cohort. Nevertheless, to support this assumption we provide sensitivity analyses comparing the trends using Ct-values and interpolated parasite load under several assumptions (see S3 Text for details).

### Evaluation of qPCR observations vs. expectations among treated individuals conditional to a single pre-treatment value

Third, we aimed to evaluate whether, over time, if Ct-values for those treated in each of the four arms of D3 would substantially deviate from the predicted Ct-values range, which was based on their baseline (pretreatment) Ct-value, here assuming that each individual pretreatment value approximated the individual steady-state. We used the observed Ct-values from D2 (i.e., longitudinal data of untreated individuals in the placebo arm) in order to first identify the minimum Ct-value (i.e., maximum observed parasitemia) for those with at least one negative value. Assuming that the individuals from the placebo arm in the D2 are a good representation of the total population evaluated in the randomized clinical trial, the threshold was interpreted as a cut-off limit (namely threshold 1) which allowed us, using a single time point observation, to roughly classify individuals in D3 into a) individuals who are likely to present only detectable parasitemia in the subsequent measurements b) individuals who are likely to present at least one sample with non-detectable parasitemia in the subsequent measurements. For each of these two groups, we could then estimate the maximum predicted range of Ct-values. In addition, we identified the minimum Ct-value (threshold 2) for those with 50% or more of negative values from the total observations using the same rationale. We performed a similar procedure for individuals in D1 as sensitivity. Last, we compared the observations per individual over time for each of the arms of the D3 against the predicted Ct-values ranges estimated in the previous step, using the baseline (pretreatment) Ct-value to assign each individual to one of the following: 1) those expected to always test positive, with at least one observation over the cut-off 2) those expected to have >50% negative values in subsequent measures 3) those expected to have <50% negative values in subsequent measures.

## Results

### Qualitative assessment of parasitological status in chronic Chagas disease patients over time

**Fig 1:**
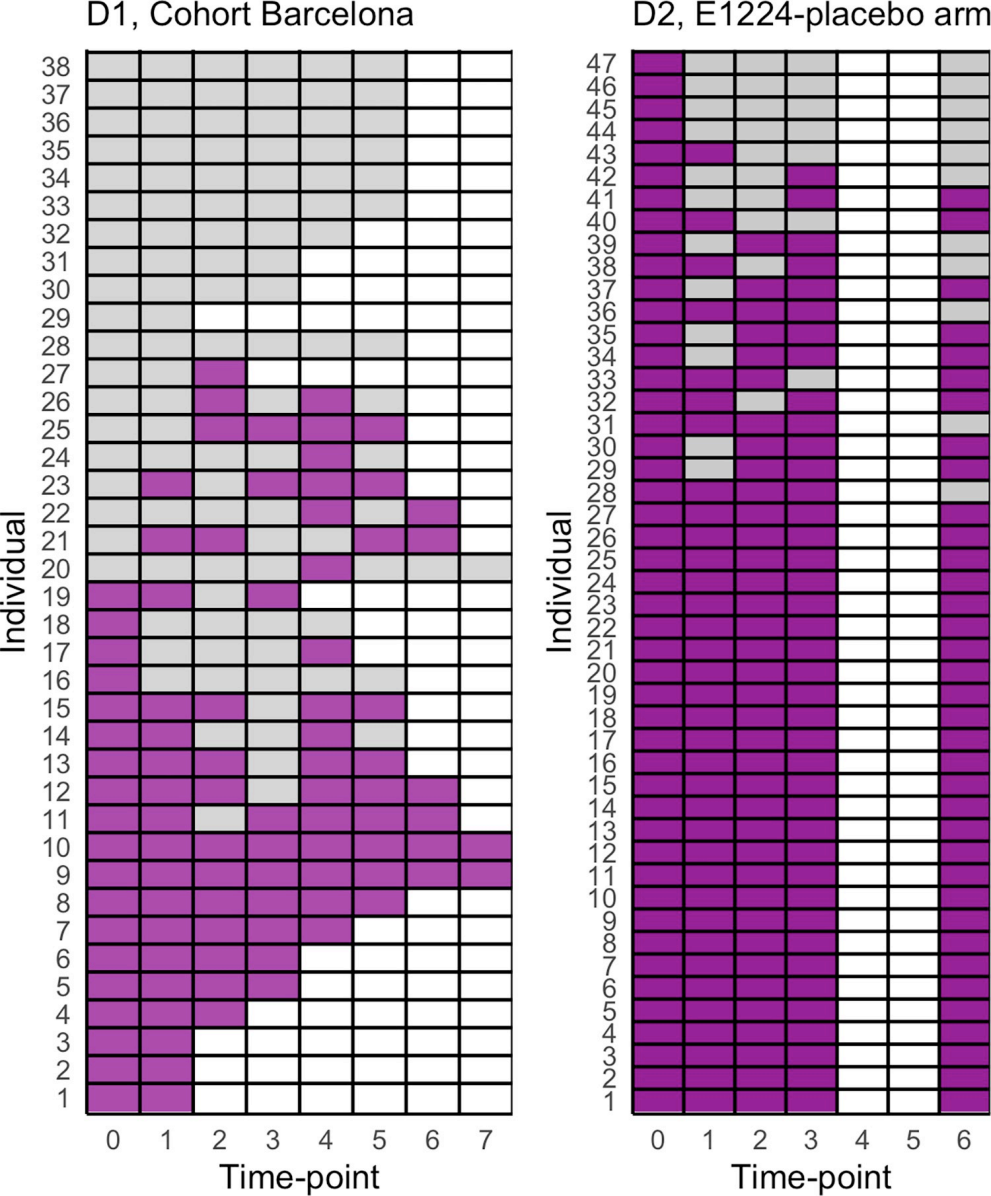
Qualitative parasite detection series by individual and dataset. Showing parasitemia at each time point per individual (as PCR positive in purple, or PCR negative in gray) for individuals in D1 (left panel) and D2 (right panel). White cells represent unavailable observations.

First, we evaluated parasite positivity at each time point for each individual included in datasets 1 and 2 (D1-D2). As seen in Fig 1, three major patterns can be distinguished: 1) individuals with continuous detectable parasitemia, 2) individuals alternating between detectable and non-detectable parasitemia, and 3) individuals with continuous non-detectable parasitemia. D2 does not include individuals with continuous non-detectable parasitemia due to the exclusion of individuals with a negative PCR result at screening in that dataset.

The results of the hidden Markov model (HMM) analysis show that a 2-category model suits the data better than the one with a single state, with significantly lower AIC/BIC computed for both D1 and D2 (see Table A in S1 Text for all computed values). Individuals are either in one category with a high probability of parasite detection (0.86 and 0.89 in D1 and D2, respectively) or in a category with a low probability of detection (0.23 and 0.13 respectively). Model estimates of transition probabilities also suggest that individuals in both datasets have an extremely low probability of transitioning from one category to the other within 2-months. The analysis supports a model in which the probability of detecting parasite DNA in samples from chronically infected individuals depends on the parasitological category individuals were in at the time of sampling.

### Quantitative assessment of parasitological status in chronic Chagas disease patients over time

**Fig 2:**
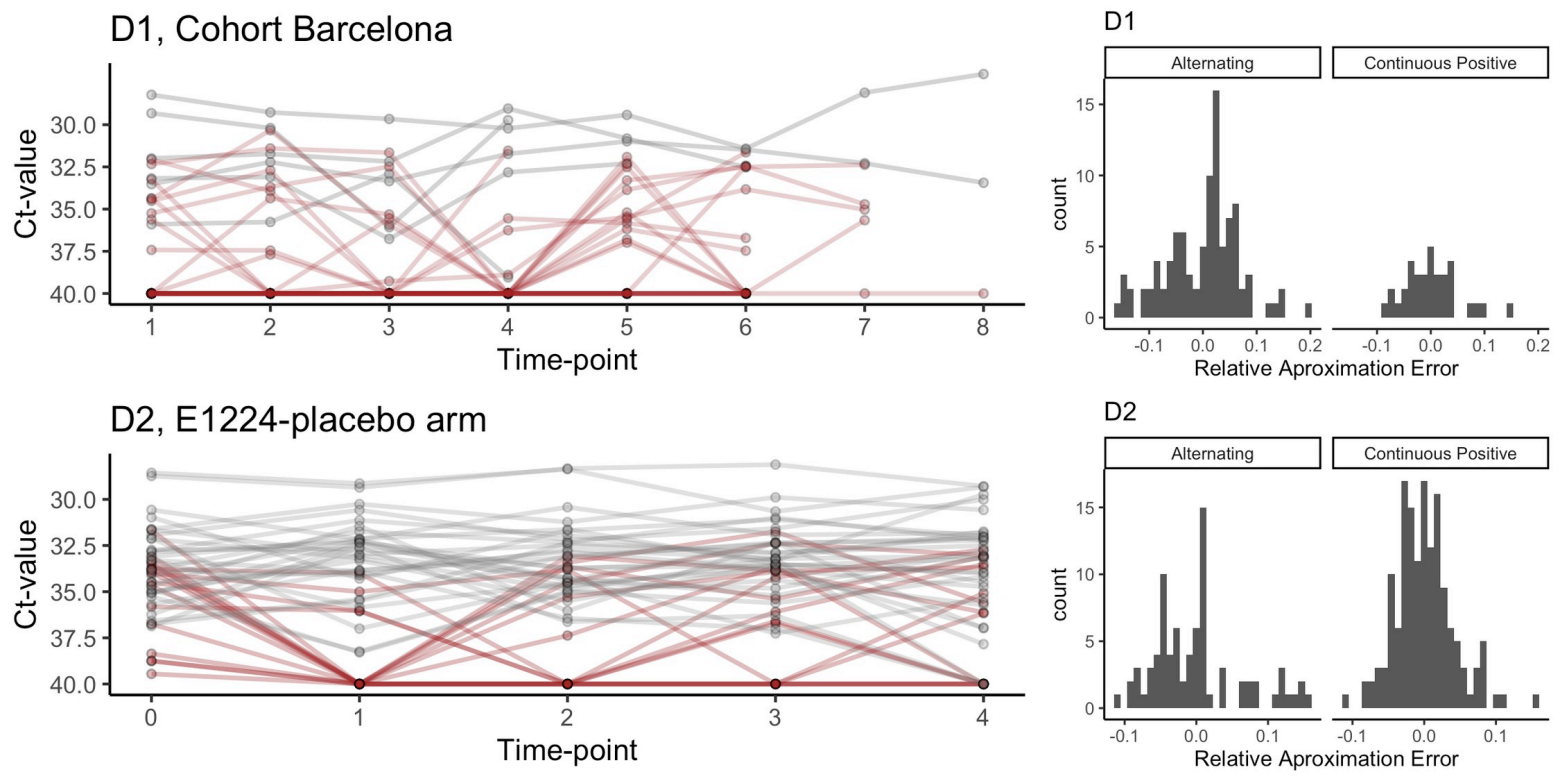
Ct-values as individual trajectories over time for both D1-D2 and percentage error distributions by category. Left row panels showing individual trajectories over time among those with always detectable parasitemia (gray) and those alternating detectable/undetectable parasitemia (red) for D1 (top) and D2 (bottom). Right panels showing the distribution of the approximation errors (as frequencies) relative to each individual series mean Ct-value and disaggregated by category, continuous detectable parasitemia on the left side vs alternating detectable/undetectable parasitemia on the right side—for D1 (top) and D2 (bottom).

Fig 2 shows individual trajectories of qPCR over time for both D1 and D2 (left panels), suggesting highly consistent cycle threshold (Ct)-values at an individual level. The histograms in Fig 2 (right panels) show the joint distribution of the relative approximation error by group and dataset. Among individuals alternating between detectable and undetectable parasitemia, the mean absolute percentage error (MAPE) is 5.4% and 4.6% for D1 and D2 respectively, and among those showing only positive results the MAPE is 3.8% and 3%. These mean percentage errors represent mean Ct-values differences ranging between +/- 1 and +/-2.5 Cts. Under the assumption that a 10-fold difference in parasite load is expected for approximately 3.3 Cts [25], our analysis shows that on average, individual Ct-values can range up to 5 to 50-fold around the steady-state when assessed every 2 months. In addition, and conditional on the time of follow-up of the datasets, individual-level Ct-values pivot around a steady state (here assumed to be represented by the median value of all individual observations), while the quantitative trajectories seem to be distributed continuously over the Ct-values range. The finding on the individual steady-state holds when using interpolated parasite load to construct the individual time series, as parasite equivalent (pEq./mL)/mL, instead of Ct-values, as shown in Figs A and B in S3 Text. Further details on the results of the sensitivity analyses can be found in S3 Text.

The smaller MAPE values for individuals in both datasets with continuous detectable parasitemia suggest that at lower Ct-values (higher parasitemia), a single observation is more representative of the mean value computed from observations over time, compared to a single observation at higher Ct-value (lower parasitemia). Given the methods applied, greater accuracy was expected for the data in D2 (i.e., less measurement noise when evaluating parasitemia by qPCR), which is consistent with the differences in the MAPE observed between datasets. Finally, when using the average of two observations (here with a 2-months gap) for D2 instead of one single time point observation, the MAPE reduced to 1.8% and 3.1% for those always showing detectable parasitemia and those alternating between detectable and undetectable parasitemia, respectively, while using the average of observations at three time points, reduced the MAPE to 1.3% and 1.8%, respectively.

### Evaluation of qPCR observations vs. expectations among treated individuals, relying on a single pre-treatment value

**Fig 3:**
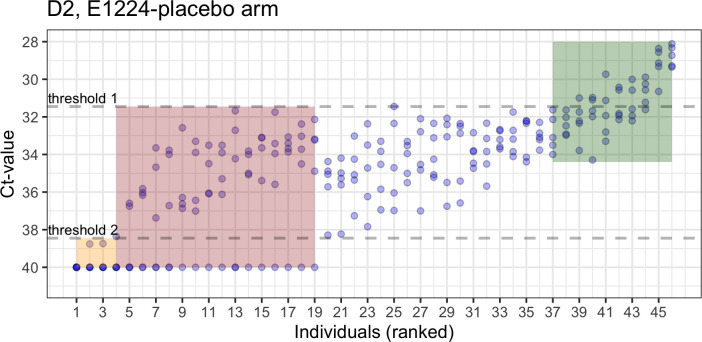
Ct-values for individuals ranked by parasitemia pattern category and mean Ct-value (from highest to lowest). The red area comprises individuals with alternating detectable/ undetectable parasitemia with >50% timepoints having detectable parasitemia, with the y-axis spanning 0 and the minimum Ct-value within the group. The green area comprises individuals who always have detectable parasitemia, with the y axis showing the Ct-value range for individuals with continuous positive detection and at least one observation above the threshold 1. The orange area comprises individuals with alternating detectable/undetectable parasitemia with <50% timepoints having detectable parasitemia, and the y-axis spanning 40 and the minimum Ct-value within the group (threshold 2).

Fig 3 shows the Ct-values obtained per individual and ranked by category and mean Ct-value among individuals in D2. Again, individual Ct-values group at levels that represent a continuum over the range. Fig 3 also shows the expected Ct ranges when there is one single observations: for those individuals with detectable/undetectable parasitemia, the range is from the truncated value of 40 to the threshold 1-value around 32 Ct, while for those with at least one single measure above the cut-off the expected range is from 33.5 to 28 Ct. In addition, of those with more than 50% negative values the range is from 40 to 38.3 Ct. Notably, when relying on one single observation, individuals with only detectable parasitemia and no Ct-value over the cut-off at baseline are indistinguishable from those with alternating negative/positive values with >50% values under 40 Ct. A subgroup of these is also expected to show values over the threshold 1-value in subsequent measures because of the imitations of the classification procedure. For D1 (see S1 Fig), the expected Ct-range for individuals with alternating detectable/undetectable parasitemia is 40–30 and approximately 32.5–28 for those with only positive results. Here, there is greater overlap for the expected ranges for Ct-values between categories compared to D2, which could be explained by the less accurate classification of individuals in either continuous positive or alternating values due to the difference in the number of qPCR replicates from samples (2 replicates out of 1 sample in D1 vs. 9 out of 3 samples in D2).

**Fig 4:**
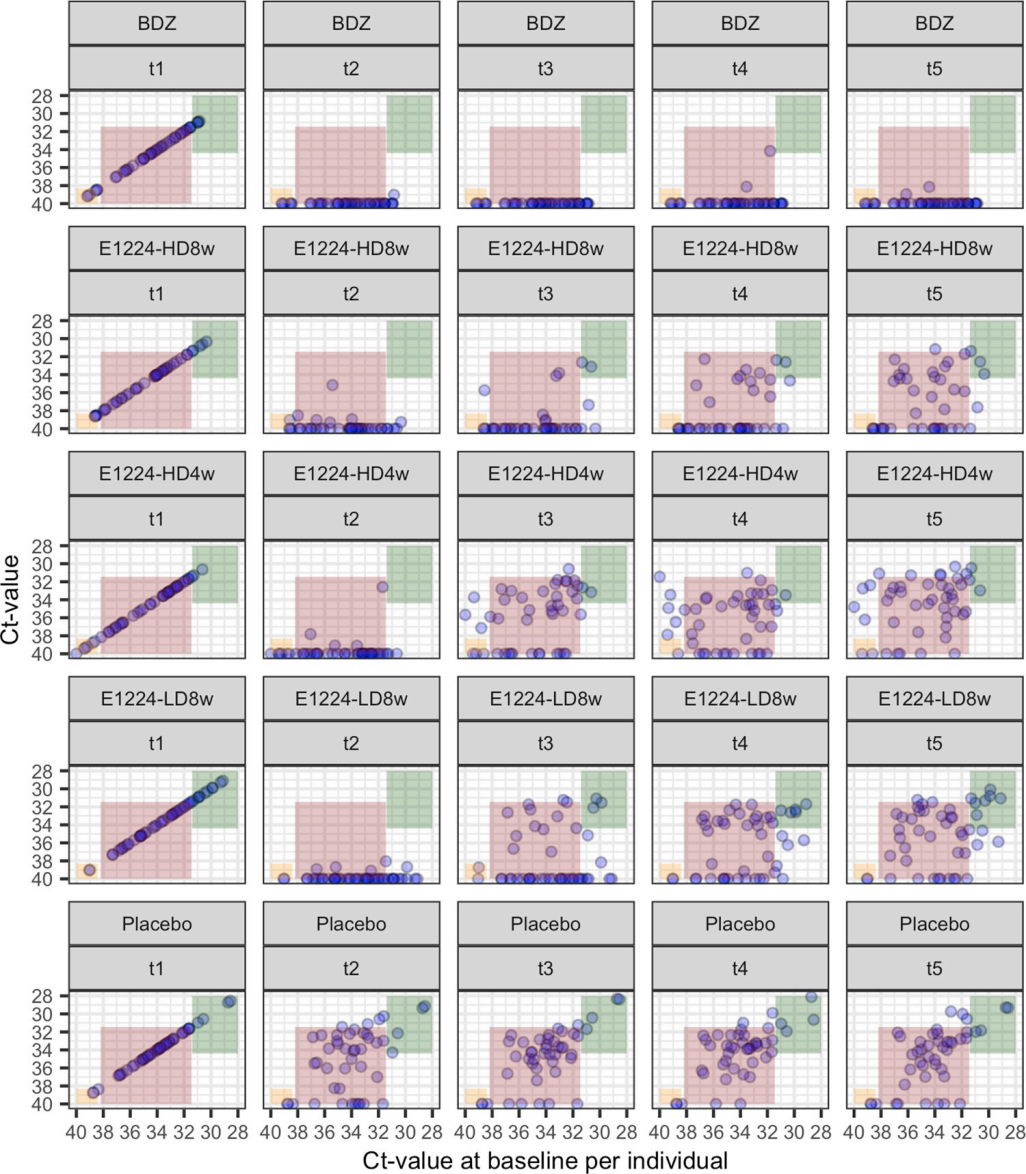
Individual Ct-values for all treated patients in the E1224-efficacy study over time against predicted Ct-range, the expected counterfactual under the non-treatment assumption. Individual Ct-values over time by trial arm and time point at each panel. The x-axis represents the individual Ct-value at baseline, and the y-axis represents the individual Ct-value at each time point assessed. Colored areas represent the expected Ct-value range under the no treatment counterfactual for BDZ, E1224-HD8w, E1224-HD4w, E1224-LD8w and placebo, at time-points t0-t4, representing baseline, 2, 4, 6, and 12 months respectively. Colored areas represent predicted counterfactual Ct-values range for a) individuals with alternating detectable/undetectable parasitemia with at least one value under threshold 2 (orange); b) individuals with alternating detectable/undetectable parasitemia with any value over threshold 2 (red); and c) individuals with continuous detectable parasitemia with at least one value over threshold 1 (green).

Last, as shown in Fig 4, the predicted Ct-ranges for subsequent measurements for individuals in D3 after a single observation (i.e, the counterfactual expectation under no-treatment) allows visualization of the deviation from true observation after treatment. The placebo arm (D2) is also shown as control. When comparing arms, the distribution of the baseline Ct values varies by arm; for example, the E1224-LD8w arms includes substantially greater number of individuals with Ct-values over the threshold 1-value, n = 10, vs. n = 3, 5, 4 and 3 in the BDZ, E1224-HD8w, E1224-HD4w, and Placebo arms respectively (note that the total number of individuals included is roughly the same in all arms). Similarly, the number of individuals with baseline Ct-values under the threshold 2-value is higher in the E1224HD4w, n = 6, compared to 3, 3, 3 and 2 in the BDZ, E1224-HD8w, E1224-LD8w, and Placebo arms respectively. Of note, the evaluation of efficacy outcomes included controlling for baseline characteristics [10]. Further, overall Ct-values among the E1224 regimens are consistent with a reduction in the individuals’ steady-states following treatment, but subsequently rising back towards pre-treatment values around the end of follow-up, a dynamic that is not seen in the BDZ arm, which is expected given the high efficacy of BDZ in clearing parasitemia [9]. Moreover, for the E1224 regimens with longer treatment (8 weeks), a proportion of those with low Ct-values (over threshold 1, high parasitemia) seem to remain at a lower steady-state at the end of follow-up: 2 out of 5 (40%) in the E1224HD8w arm and 3 out of 10 (30%) in the E1224LD4w. Strikingly, for the E1224HD4w regimen with the shortest treatment time, during follow-up of individuals with very high Ct-values at baseline (i.e., over the threshold 2, corresponding to very low parasitemia) there was an increase towards the values expected for those with lower Ct-values at baseline, suggesting this regimen increased the individuals’ steady state parasitemia level, following treatment, for the whole follow-up period.

## Discussion

In this study, we evaluated a unique longitudinal *T*.*cruzi* qPCR data set of individuals with chronic Chagas disease using a novel statistical inference framework. Our analysis is consistent with untreated individuals characteristically exhibiting a steady-state parasitemia. Levels of parasite DNA detected by qPCR fluctuate around the steady-state when sampled at two-months gap over one year of follow-up, even though individual-level steady states are distributed over a wide range of Ct-values. In contrast, current approaches using group-level, cross-sectional and/or qualitative interpretations of PCR provide less information on parasitological dynamics in chronically infected individuals.

This is the first systematic evaluation of individual *T*.*cruzi* qPCR dynamics over time in humans with chronic Chagas disease. Nevertheless, Cerisola et al. [29] had previously evaluated parasitemia over time using xenodiagnosis with the overall findings aligned with our study, with individuals having a parasitemia steady-state over time. While our analysis is consistent with the qualitative interpretation that *T*.*cruzi-*infected individuals might have either positive or negative PCR results depending on the accuracy of the molecular method, we found that interpretations from a single time-point assessment can be substantially biased given the subsequent dynamics if not properly addressed. For example, a large proportion of individuals in our study had a steady-state close to the limit of detection and thus, after a single positive or negative result, any following assessment -here assessed at 2 months intervals- was frequently different. Furthermore, we found that when the range of Ct-values at inclusion is not equally distributed between the arms of a clinical trial, the interpretation of efficacy using qPCR might be biased. These findings, while not necessarily invalidating previous analysis, have direct implications for the interpretation of drug-efficacy trials if individuals are classified PCR-positive or negative following a pretreatment assessment using only one single time point [18]. Such a misclassification of a proportion of patients regarding their parasitological status could also explain the discrepancy between PCR results and severe forms of the disease, such as chagasic cardiomyopathy [18] or digestive damage [19], in clinical evaluations. With regards to optimizing the design of future trials, our analysis suggests that performing qPCR to assess Ct-values at two pretreatment time points 2-months apart would substantially improve the classification of the individual’s steady-state, while limiting the burden of blood withdrawn to the patients. Notably our results confirm that taking 3 samples per time point as in the protocol used for D2-D3 (see Materials and Methods) lead to smaller MAPE than that of D1 (which uses one single sample) suggesting that the previous reduced the uncertainty in estimating the individual’s steady-state from single time point assessments. However, the differences between the dataset populations and inclusion criteria could confound such interpretation.

Individual-level quantitative evaluation of pre- and post-treatment PCR outcomes can identify a reduction in the individual’s steady-state during follow-up, particularly under suboptimal treatments and among those with highest parasitemia (lowest Ct-values). While these individuals remain by definition uncured (which is nevertheless uncommon for currently available drugs in clinical practice), further research needs to be done to elucidate the clinical consequences of this reduction, as well as how long this reduction might last. For example, under the assumption that substantial changes in blood parasite load after treatment (i.e., reduced steady-state) represent an overall reduction of infection burden (i.e., a reduction in the tissue-specific parasite loads, or in the strength of the immune response), which in turn might influence disease development, several courses of treatment spaced out over time might be evaluated, aiming for the reduction of disease progression or eventually definitive parasitological cure. Interestingly, our analysis framework allowed us to identify a subgroup of individuals with very low-level steady state parasitemia that seemed to increase their steady-state parasitemia when treated with a short regimen. This potential boosting of parasitemia requires further assessment, as well as whether it might have any clinical impact. Lastly, our framework provides a rationale for disaggregating the evaluation of qPCR-based endpoints by subgroups defined by their steady-state, such as those with low Ct-values (continuous detectable high parasitemia) or those with alternating detectable/undetectable parasitemia with Ct-values that are very close to the limit of detection. As the challenges for interpreting the parasitological outcomes are different for these particular groups, it might be necessary to address them with different trial designs and/or aims. For example, in the first group, the framework allows identification of the reduction in steady-state, which makes it an important case-study for the effect of suboptimal treatment and its implications on disease development. The second group of individuals, which have been probably underrepresented in clinical trials as negative PCR at screening is typically an exclusion criterion, might also benefit from alternative approaches, such as a more intense sampling strategy, or evaluation of specific antibody seroconversion after treatment. Also, different treatment regimens can be applied according to the individual’s steady state. For example, longer treatment regimens for those with lower Ct-values (higher parasitemia) might improve cure evaluation outcomes, while individuals with higher Ct-values pretreatment might benefit from shorter regimens.

Our analysis has some important limitations. Quantitative PCR values from blood samples are assumed to represent the parasite load in the bloodstream. Because PCR targets can present several copies in one single parasite, Ct-values might not represent similar parasite loads in different individuals. This is particularly important when evaluating jointly individuals that might be infected with different discrete typing units (DTUs). However, our analyses using Ct-values or interpolated parasite load based on standardized curves (see S3 Text) focus firstly on the relative changes of the individual level trends, which hold if individuals are not reinfected over time. Additionally, multiclonal infections in the same individual can further bias comparison of the PCR results between individuals, but also at individual level in the case of multiclonal infections with different drug susceptibility or reinfections during follow-up, the last a possible scenario for D2-3. This has been hypothesized previously [18]. Furthermore, the use of procedures with lower detection accuracy, as in D1 using two qPCR replicates instead of 9 in D2, impacts the calculation of the threshold values and in turn the uncertainty of the prediction of the Ct-ranges. Also, we did not evaluate immune response over time. A better understanding of these issues is key and needs to be included in future work. Nevertheless, our interpretation of the individual-level steady-state in untreated individuals would hold, under the assumption that within-host parasite genetic diversity is constant during the follow-up if individuals are not reinfected. The interpretation of our analysis is also limited by the design frameworks of the datasets, such as the fact that: a) we could only evaluate measurements at intervals of 2-months; hence, evaluation of smaller differences within timepoints (such as 2 weeks or one month) may optimize the assessment; b) inclusion criteria for D2-D3 required a positive PCR at screening, which probably reduced the representation of those with only undetectable parasitemia during follow-up, and c) pretreatment classification for D3 was based on a single time point assessment, resulting in higher uncertainty regarding their steady-state. Overall, further confirmation of our findings would benefit from considering these limitations.

Despite of these limitations, we propose a novel framework to improve the understanding of the dynamics of blood parasitemia over time in adult patients with chronic Chagas disease, including the following recommendations for the design of novel drug-efficacy trials: 1) estimate individual-level steady-states quantitatively, using at least two pretreatment time points with a 2-months gap between them, while maximizing the diagnostic accuracy of the method 2) assess the homogeneous distribution of the pretreatment individual-level steady-states between arms, 3) adjust efficacy endpoints, such as absence of detectable parasitemia during follow-up, depending on the pretreatment steady-state; and 4) evaluate individual-level steady-state reduction after treatment, and potential subsequent clinical, parasitological, or immunological assessments. Furthermore, to overcome the limitations arising from evaluating multiclonal infections and populations affected with different DTUs, direct identification of DTUs from blood samples of individuals in clinical trials, which is currently limited to high load parasitemia [14], as well as longitudinal evaluation of with-in host multiclonal parasite diversity in Chagas cohorts, would help to disentangle potential differences in clinical response and parasite dynamics. We conclude that embedding individual-level, quantitative analysis of parasitemia over time within drug-efficacy studies and studies evaluating disease severity and progression is essential to recognizing differences in parasitological status between participants, adjusting outcomes to reduce potential bias, and optimizing and redesigning research to effectively reduce the burden of Chagas disease.

## Supporting information

S1 Text Details on the datasets and diagnostic procedures. (PDF)

S2 Text Statistical details on the Hidden Markov Model. **Table A in S2 Text.** Parameter estimates for M1 and M2 in D1-2 using the Expectation-Maximization algorithm. **Fig A in S2 Text.** Schematic representation of evaluated M1-M2 Hidden Markov Models.
(PDF)

S3 Text Sensitivity analyses for the use of Ct-values compared to interpolated parasite load. **Fig A in S3 Text.** Sensitivity analyses for the Barcelona Cohort, D1. Left row panels showing individual trajectories over time among those in D1 with always detectable parasitemia (gray lines) and those alternating detectable/undetectable parasitemia (red lines) when using Ct-values (top), using interpolated parasite loads (as pEq./mL) with approach 1 (middle top), using interpolated parasite loads with approach 2 (middle bottom) and using interpolated values adjusted for sample volume, approach 3 (bottom). Right panels showing the relationship between Ct-values and parasite loads for a given interpolation approach for individuals with always detectable parasitemia (gray dots) and those alternating detectable/undetectable parasitemia (red dots). **Fig B in S3 Text.** Sensitivity analyses for the E1224-Placebo arm, D2. Left row panels showing individual trajectories over time among those in D2 with always detectable parasitemia (gray lines) and those alternating detectable/undetectable parasitemia (red lines) when using Ct-values (top), using interpolated parasite loads (as pEq./mL) with approach 1 (middle) and using interpolated parasite loads with approach 2 (bottom). Right panels showing the relationship between Ct-values and parasite loads for a given interpolation approach for individuals with always detectable parasitemia (gray dots) and those alternating detectable/undetectable parasitemia (red dots). Log 10 scale is used for the y-axis to facilitate visualization. **Fig C in S3 Text.** Distribution of Ct-values relative to sample volume in the Barcelona Cohort
(PDF)

S1 Fig Ct-values of individuals in D1 ranked by parasitemia pattern category and mean Ct-value (from highest to lowest). The red area represents individuals with alternating detectable/ undetectable parasitemia with >50% timepoints having detectable parasitemia, with the y-axis spanning 0 and the minimum Ct-value within the group. The green area represents individuals who always have detectable parasitemia, with the y axis showing the Ct-value range comprising that of individuals with continuous positive detection and at least one observation over the threshold 1. The orange area spans individuals with alternating detectable/ undetectable parasitemia with <50% timepoints having detectable parasitemia, and the y-axis spanning between 40 and the minimum Ct-value within the group (threshold 2).
(TIF)
