# Supplementary material for: Human Trypanosoma cruzi chronic infection leads to individual level steady-state parasitemia: Implications for drug-trial optimization in Chagas disease
Source: PLoS Negl Trop Dis. 2022 Nov 21;16(11):e0010828. doi: 10.1371/journal.pntd.0010828 (PMC9721471; doi:10.1371/journal.pntd.0010828)
Supplement: S1 Text — (PDF) [file pntd.0010828.s001.pdf]

S1 Text. Details on the datasets and diagnostic procedures

Dataset 1

Individuals diagnosed with Chagas disease by serology at the Vall d'Hebron University Hospital (Spain) were followed up to monitor disease development. Patients were asked to come regularly to the Infectious Disease outpatient clinic until treatment was available. Once the drug became available, patients were treated according to national guidelines. Clinical visits included baseline systematic assessment (physical examination, chest x-ray, esophagogram and electrocardiogram) and blood withdrawal for performing quantitative (q)PCR. Further physical examination and qPCR was performed at each follow-up visit, approximately every 2 months. The State department of origin prior immigration for the Bolivian cohort comprised 48.5% (18/37) of patients from Cochabamba, 37.8% (14/37) of patients from Santa Cruz and 13.5% (5/37) of patients from Chuquisaca.

A database was then constructed using the qualitative results of the qPCR (see below) as categorical variables ("positive" or "negative" detection of *T.cruzi* DNA) and included all patients attending the clinic during the study period with at least 2 samples from consecutive visits. The D1 accounted for 38 sequences (from a similar number of patients) with a total of 213 observations. The individual sequence size ranged from 2 to 8 observations, 32 of which had 4 or more consecutive time point observations. From this subset, 3 showed consistently "positive" detection and 12 consistently undetectable DNA. The remaining 18 sequences showed alternation between detectable and undetectable parasitemia.

*Serological diagnosis***.** Chagasic patients were diagnosed using serology and the concordance between two enzyme linked immunosorbent assays (EIA; OD > 0.9) following WHO guidelines. One EIA was based on a recombinant *T. cruzi* antigen (Bioelisa Chagas. Biokit. Lliçà d'Amunt, Spain) and the other on whole lysate antigen EIA (ORTHO *T. cruzi* ELISA Test System, Johnson and Johnson, High Wycombe, United Kingdom).

Quantitative PCR procedure. All qPCR procedures were standardized to ensure uniformity of methodology and procedure across the study as described here. For each repeat blood sample from the patient cohort, 2-10 ml blood was drawn into a tube containing EDTA and pre-treated with a 1:1 volume of guanidine hydrochloride 6M (Sigma Aldrich) for 24 hours at room temperature. DNA extraction was performed using 200 *µ*l of blood-guanidine solution with silica membrane technology (NucliSens easyMAG. Biomerieux. France) and eluted in 50 *µ*l of elution buffer according to the manufacturer's instructions. Five *µ*l of extracted DNA was used for qPCR, each reaction was performed at least in duplicate and triplicate reactions were performed where appropriate. Quantitative-PCR assays were based on amplifying the *T. cruz* 166 bp satellite DNA locus with the primers Cruzi-1 (ASTCGGCTGATCGTTTTCGA) and Cruzi-2 (AATTCCTCCAAGCAGCGGATA) and the TaqMan probe Cruzi 3 (5´-FAM-CACACACTGGACACCAA-3´- MGB) as previously described [1]. An internal amplification control reaction was used for each qPCR assay using the RNase P human gene locus to validate the efficiency of each *T. cruzi* qPCR reaction (Taq Man Human RNase P detection System, Applied Biosystems). An established panel of DNA extracted from the whole blood of

Chagas disease patients was used as qPCR controls providing 4 positive controls for high and low parasitaemia and a panel of negative controls to assess contamination. The qPCR master mix comprised 1X QuantiTec Multiplex PCR NoRox kit (Qiagen), 0.1 X "RNase P gene" detection reagents, 400 nM each primer Cruzi 1 and Cruzi 2 and 100 nM for the probe Cruzi 3. Each qPCR reaction was performed in duplicate in a 25 µl reaction. The samples were subject to a real time thermocycle program (CFX96 Real Time System, Biorad), viz. 15 min at 95 ºC and 45 cycles of 15 sec at 95 ºC and 1 min at 58 ºC. Q-PCR amplification was considered valid when the qPCR of the human RNase P internal control was within the normal range specified in the manufacturer's instructions. A qPCR reaction of a patient sample was considered positive for *T. cruzi* when at least one cycle threshold (Ct) for the *T. cruzi* targets (Cruzi 1 and Cruzi 2) was < 40, and negative when both Ct were ≥ 40. The qPCR procedure was validated by a blind qPCR of *T. cruzi* spiked blood provided in 4 panels of 12 aliquots per panel by an external reference laboratory. A patient was considered PCR positive if at least one qPCR replicate showed detectable parasitemia. The average cycle threshold (Ct) value of all measurements below 40 was used for each time point (up to 2 qPCR replicates in D1).

For sensitivity analyses, Ct results were interpolated against a qPCR standard curve to obtain the number of parasites per ml of patient blood, and the concentration of parasites per aliquot was varied to assess the robustness of the in-house qPCR assay. Specifically, the standard curve was constructed using a 5 ml blood sample inoculated into a tube containing EDTA from a *T. cruzi* seronegative individual. The blood sample was spiked with 10^5 parasites ml^{-1} of *T. cruzi* epimastigotes from the Maracay strain, DTU TcI and immediately mixed with one volume of guanidine hydrochloride 6M. The guanidine, EDTA and blood mixture was serially diluted 10-fold to cover a range between 10^5 and 10^1 parasite ml^{-1} in addition to one 2-fold dilution to achieve 5 parasites ml^{-1}. Total DNA was then extracted as described above to assess the dynamic range of the real-time PCR technique as a standard curve. The copy number of the 166 bp satellite-DNA is approximately one logarithm with base 10 higher in DTU TcII, TcV and TcVI compared with TcI, the reference strain [2]. In Bolivian infected individuals, TcV predominates [3], but also TcII and TcVI. Thus, the interpolation of the *T. cruzi* qPCR Ct for parasites ml^{-1} was adjusted for the 1 log expected difference between the clinical samples and the strain used for standardization. The lower cut-off for the empirical interpolation in the original clinical assessment for all experiments in this dataset yielded a value of 37.5 cycle-threshold, which corresponds with 4 pEq./10 mL after adjusting for DTU satellite DNA density. For the sensitivity analysis testing whether sampling volume might affect Ct-values, the interpolated parasite load was further adjusted by the sampled volume of blood.

Datasets 2 and 3:

Details on the dataset and diagnostic procedures can be found at Gascon et al [4] and Melo et al [5]. Briefly, individuals aged 18–50 years and weighing >40 kg were confirmed for diagnosis of *Trypanosoma cruzi* infection by at least two of three conventional serological tests and serial positive qualitative PCR (at least one of three samples collected over 7 days). Included participants required a normal screening electrocardiogram (ECG) at screening, and no contraindication to the study drugs. Women should have not been pregnant or breastfeeding and should have been on contraception for at least 4 months. Participants who had signs or

symptoms of chronic Chagas disease, acute or chronic health conditions, abnormal laboratory tests, had received prior benznidazole, nifurtimox or systemic treatment with azoles, allopurinol, or any concomitant antimicrobial and immunosuppressant agents or had history of alcohol abuse or other drug addiction were excluded. Quantitative PCR was performed 3 times in 3 different samples per time point, 2 on the same day and one 7 days later; time points for our analysis included baseline (before treatment), and 10 weeks, 4, 6 and 12 months after treatment [3].

*Serological diagnosis***.** Two EIAs were used, based on recombinant and crude antigens respectively: Chagatest ELISA recombinante, Wiener Lab, Rosario, Argentina, and Chagatest ELISA lisado, Wiener Lab.

Quantitative PCR procedure. Samples consisted of peripheral blood mixed with an equal volume of 6 M guanidine hydrochloride, 0.2 M EDTA, pH 8.0, buffer. The High Pure PCR template preparation kit (Roche Diagnostics Corp., Indianapolis, IN) was used to process 300 µl of each guanidine-EDTA-blood sample, and DNA was eluted in 100 µl elution buffer, as previously described [2]. Quantification of *T. cruzi* satellite DNA was performed using a multiplex TaqMan real-time quantitative PCR and an internal amplification control in a single-tube reaction was performed. A duplex qPCR targeted to *T. cruzi* satellite DNA (SatDNA) and an internal amplification control were used as previously described [2]. The qPCR reactions were carried out with the use of FastStart universal probe master mix (Roche Diagnostics GmbHCorp., Mannheim, Germany) with 5 µl DNA extract in a final volume of 20 µl. Cycling conditions were a first step of 10 min at 95°C, 40 cycles at 95°C for 15 s, and a final step of 1 min at 58°C. The amplifications were carried out in a Rotor-Gene Q (Corbett LifeScience, Cambridgeshire, United Kingdom) real-time PCR device. Each PCR experiment included positive and negative controls. External quality control panels for PCR were evaluated during the study period, using blinded seronegative blood samples spiked with serial dilutions of cultured *T. cruz*i. PCR positivity was defined as a positive result in at least one of the 3 replicates of each of the three different samples. The median cycle threshold (Ct) value of all measurements below 40 was used for each time point (up to 9 qPCR replicates).

For quantification purposes, standard curves were plotted with 1/10 serial dilutions of total DNA obtained from a guanidine-EDTA blood seronegative sample spiked with 10^5 parasites ml^{-1} LL014-1-R1 Cl1 *T. cruzi* stock (TcV) cultured epimastigotes. One negative control and two positive controls containing 10 and 1 fg/µl *T. cruzi* CL-Brener DNA were included in every run [6]. The lower cut-off for empirical interpolation in the clinical assessment for all experiments in these datasets yielded a value of 35 cycle-threshold, which approximately corresponds with 1 pEq /10 mL.

References

1. Piron M, Fisa R, Casamitjana N, López-Chejade P, Puig L, Vergés M, et al. Development of a real-time PCR assay for Trypanosoma cruzi detection in blood samples. Acta Trop. 2007;103: 195–200.
2. Duffy T, Cura CI, Ramirez JC, Abate T, Cayo NM, Parrado R, et al. Analytical performance of a multiplex Real-Time PCR assay using TaqMan probes for quantification of Trypanosoma cruzi satellite DNA in blood samples. PLoS Negl Trop Dis. 2013;7: e2000.
3. Parrado R, Ramirez JC, de la Barra A, Alonso-Vega C, Juiz N, Ortiz L, et al. Usefulness of Serial Blood Sampling and PCR Replicates for Treatment Monitoring of Patients with Chronic Chagas Disease. Antimicrob Agents Chemother. 2019;63. doi:10.1128/AAC.01191-18
4. Torrico F, Gascon J, Ortiz L, Alonso-Vega C, Pinazo M-J, Schijman A, et al. Treatment of adult chronic indeterminate Chagas disease with benznidazole and three E1224 dosing regimens: a proof-of-concept, randomised, placebo-controlled trial. Lancet Infect Dis. 2018;18: 419–430.
5. Melo MF, Moreira OC, Tenório P, Lorena V, Lorena-Rezende I, Júnior WO, et al. Usefulness of real time PCR to quantify parasite load in serum samples from chronic Chagas disease patients. Parasit Vectors. 2015;8: 154.
6. Burd EM. Validation of laboratory-developed molecular assays for infectious diseases. Clin Microbiol Rev. 2010;23: 550–576.
