# Supplementary material for: Human Trypanosoma cruzi chronic infection leads to individual level steady-state parasitemia: Implications for drug-trial optimization in Chagas disease
Source: PLoS Negl Trop Dis. 2022 Nov 21;16(11):e0010828. doi: 10.1371/journal.pntd.0010828 (PMC9721471; doi:10.1371/journal.pntd.0010828)
Supplement: S2 Text — Table A in S2 Text. Parameter estimates for M1 and M2 in D1-2 using the Expectation-Maximization algorithm. Fig A in S2 Text. Schematic representation of evaluated M1-M2 Hidden Markov Models. (PDF) [file pntd.0010828.s002.pdf]

S2 Text. Statistical details on the Hidden Markov Model

Let us consider the observations with categorical realization *i*= 0,1 (non-detection or detection of parasite DNA) as a vector of individuals (N=1..j) with evenly distributed timepoints (t=0...k). We have then a set of independent sequences $Y_{j,k}$ made with a binary probability distribution $\pi_{p,q}$. Under the assumption that observations depend only on the probability of detection, this can be modeled (M1) as a single-state ($X = 1$) process generating the independent binomial distribution probability $\pi_{0,1}$ with parameters p_1 and q_0=*1*-$p_{1.}$. We consider this our baseline model which accounts for the current interpretation of parasite DNA detection in chronic Chagas.

Alternatively, we can model $X > 1$ unobserved states X= x_a..x_b...x_n, changing in time according to a Markov process. The Markov property, where the realization of X_t only depends on X_{t-1} is assumed for the latent state process. The underlying latent state sequence between $x_a$and x_b is defined by the transition probabilities $\Gamma = \gamma_{a,b}$, where:

$$\gamma_{i,j} = Pr\left(X_t = x_a \mid X_{t-1} = x_b\right)$$

We assume that state transition probabilities are constant over time i.e. homogeneous. The observations are then generated from the *n* state-dependent Bernoulli distributions $\pi_1 \ldots . \pi_n$ that determine the output probability

$$Y_t | X_{t,n} = \pi_n$$

The simplest model possible consists of two latent states (M2). The single-state model M1, can be considered as a particular case of M2 with transition probability $\gamma_{i,j}$= 0 and all cases initially in state i . We can estimate the most probable values of the transmission and the emission matrix given the observations and evaluate whether M1 or M2 better explain our data. We can obtain the likelihood (L) of a sequence of observations as:

$$L(\theta|y_1, y_1 .. y_n) = \delta P(y_1)\Gamma P(y_2) \ldots \Gamma P(y_n)1$$

Where θ is the vector representing the set of parameters to estimate, δ is the stationary distribution of the Markov chain and $P(y_n)$is the probability mass function:

$$Pr(Y_t = y_{t,n} \,|\, X_t = x_{t,n}) = \pi_n \ (p_n, 1 - p_n)$$

The joint log-likelihood is then the sum of the log-likelihoods of all individual-sequences. Transition and emission probabilities estimates were obtained using the Expectation-Maximization algorithm after fitting a one vs. two-state Bernoulli-HMM using MLE [1]. Censored observations such as those at 8 and 10 months in D2 are dealt with by directly estimating the parameters of the transition and emission probabilities through maximizing the log likelihood of the dataset through the Expectation and Maximization iterations under the missing-at-random assumption [2]. Model selection was performed after computing AIC and BIC [3].

References:

1. Zucchini W, MacDonald IL, Langrock R. Hidden Markov models: definition and properties. Hidden Markov Models for Time Series. 2017. pp. 29–46. doi:10.1201/b20790-2
2. Little RJA, Rubin DB. Statistical Analysis with Missing Data. 2002. doi:10.1002/9781119013563
3. Dridi N, Hadzagic M. Akaike and Bayesian Information Criteria for Hidden Markov Models. IEEE Signal Processing Letters. 2019. pp. 302–306. doi:10.1109/lsp.2018.2886933

Fig A. Schematic representation of evaluated M1-M2 Hidden Markov Models

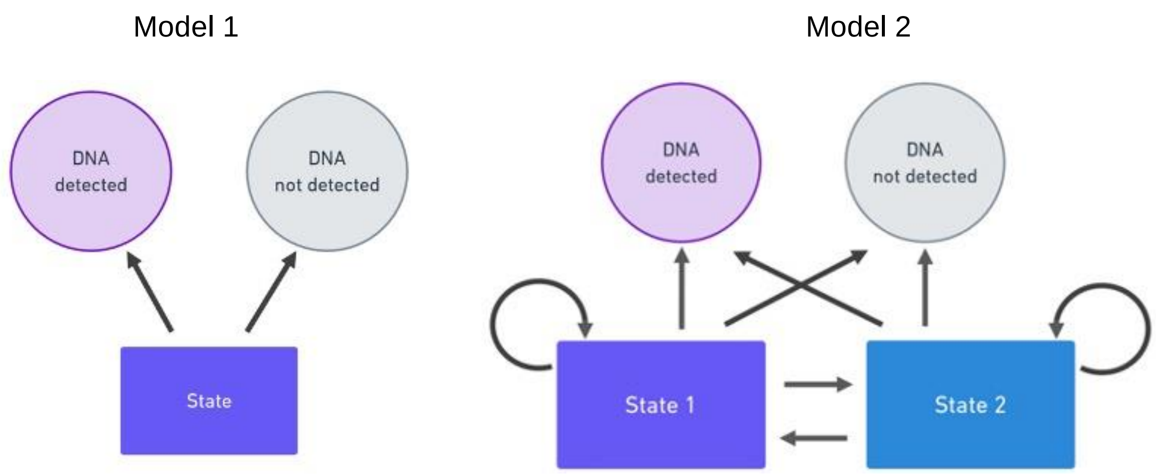

Table A. Parameter estimates for M1 and M2 in D1-2 using the Expectation-Maximization algorithm

Model and Dataset	Initial proportions	S1 to S2	S1 to S2	PCR+|S1	PCR+|S2	Maximum joint log-likelihood	AIC/BIC
M1; D1, 1 sample per time point	-	-	-	0.54	-	-106	214/215
M2; D1, 1 sample per time point	0.63-S1/ 0.36-S2	0	0	0.86	0.23	-62	134/139
M1; D2, 3 samples per time point	-	-	-	0.52	-	-195	392/394
M2; D2, 3 samples per time point	0.85-S1/ 0.15-S2	0	0	0.89	0.13	-80	171/180
