# Supplementary material for: Human Trypanosoma cruzi chronic infection leads to individual level steady-state parasitemia: Implications for drug-trial optimization in Chagas disease
Source: PLoS Negl Trop Dis. 2022 Nov 21;16(11):e0010828. doi: 10.1371/journal.pntd.0010828 (PMC9721471; doi:10.1371/journal.pntd.0010828)

S3 Text. Sensitivity analyses for the use of Ct-values compared to interpolated parasite load

To evaluate the parasitemia trends at individual level in both cohorts, we constructed the time series using median estimates of the Ct-values obtained in 2 (D1) or up to 9 (D2-D3) replicates for a single time point, based on the assumption that median estimates approximate the true parasite load. Using Ct-values facilitates the statistical analysis performed separately for each cohort, providing a biological interpretation given the log linear correlation between Ct-values and parasite loads when focusing on individual level parasitemia trends, while not requiring further assumptions than the one stated before. However, to evaluate whether our interpretation of the findings holds when using parasite loads, we convert the Ct-values into parasite concentrations interpolated from the standardized curves. For D1, the standardization curve was obtained using a TcI strain (see Section S2 for further details) therefore we corrected the parasite load by 1 log given Bolivian individuals are expected to be infected mostly with TcV/TcII/TcVI strains which approximately show a 10-fold higher number of copies of targeted DNA per parasite [1]. For the D2-D3 cohort, a standardized curve was performed using a TcV stock (see S2 Text for further details) and therefore correction for the number of targeted copies was not performed. However, Ct-values estimates over the cut-off of reliable quantification (37.5 in D1 and 35 in D2) might not be interpolated. We then approached interpolation using 2 different procedures. For the first approach, we assumed that the expectation of the distribution of the single replicate Ct-values over the cut-off approximated the parasite load with a similar relation (i.e., log-linear) than those under the cut-off. Therefore, parasitemia can be interpolated from the modeled standard curve beyond the range of the empirical estimates (stronger assumption). In this case, the limit of quantification is set to that interpolated from the maximum Ct-value considered to represent a true detection of parasites in blood DNA (i.e., < 40 Cts), which approximately corresponds with a 1 pEq/ 10 mL for Dataset 1 and 0.1 pEq/10 mL for Dataset 2-3. Second, we assumed that Ct-values over the quantification limit are truncated and thus assigned them ½ of the value of the limit of quantification, which is a common statistical procedure [2]. In this second case, part of the statistical information for individuals with Ct-values over the quantification limit is lost. Last, to test the hypothesis that sample volume in D1, where volumes ranged between 2 and 10 mL does not necessarily affect the parasite load conditional it is sufficient for DNA extraction (200 microL) we adjusted the parasite load after interpolation using the first approach and the adjusted for the individual sample volume for each replicate, testing a strong assumption. Further, we evaluated the distribution of Ct-values relative to sample volume and individual level parasite trends disaggregated by those with continuous positive replicates, those alternating and those with continuous negative replicates.

As seen in Fig A and Fig B, left column panels, representation of parasitemia over time using interpolated parasite loads, either not truncating (approach 1) or truncating (approach 2) does not lead to different trends at individual levels, with parasite loads pivoting around a steady state in the logarithm with base 10 scale, consistent with the Ct-values approach (top left panels) in both datasets. When representing the Ct vs. parasite load (right panels), while as expected the approach 1 leads to the log linear correspondence, the approach 2 truncating parasites load under the quantification limit and assigning ½ of the lowest quantifiable value tend to overestimate the parasite load relative to the approach one in both cohorts. Further, adjusting

for volume seems to artificially disaggregate parasite load estimates into two different linear relationships. This is due to an interesting distribution of the sample volumes, as seen in Fig C, where values tend to aggregate either around 2.5 mL or 6 mL. Fig C also shows that Ct-values seem randomly distributed within the two sample groups, as well as there is no evidence of correspondence between sample volume and individual level parasite dynamics. This is consistent with the assumption that sample volume does not affect Ct-value nor parasite load estimates.

References

1. Duffy T, Bisio M, Altcheh J, Burgos JM, Diez M, Levin MJ, et al. Accurate real-time PCR strategy for monitoring bloodstream parasitic loads in chagas disease patients. PLoS Negl Trop Dis. 2009;3: e419.

2. Melo MF, Moreira OC, Tenório P, Lorena V, Lorena-Rezende I, Júnior WO, et al. Usefulness of real time PCR to quantify parasite load in serum samples from chronic Chagas disease patients. Parasit Vectors. 2015;8: 154.

Fig A. Sensitivity analyses for Barcelona, D1

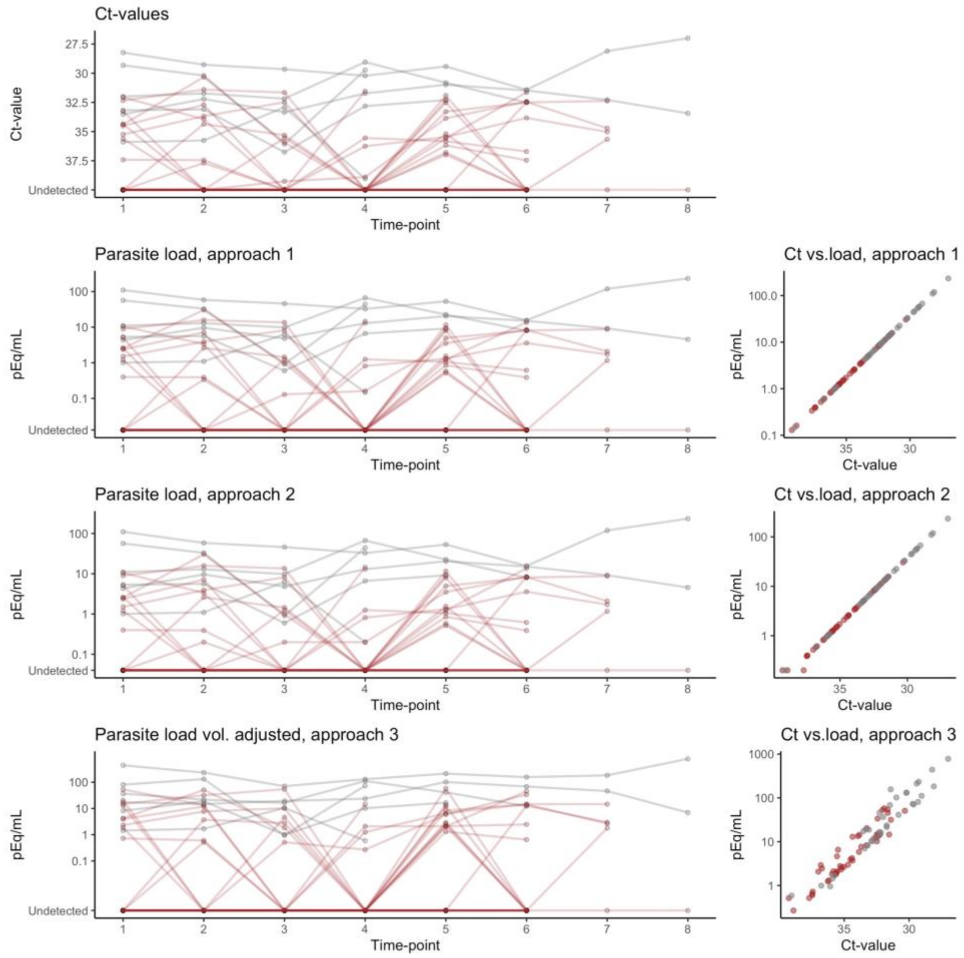

Fig A. Left row panels showing individual trajectories over time among those in D1 with always detectable parasitemia (gray lines) and those alternating detectable/undetectable parasitemia (red lines) when using Ct-values (top), using interpolated parasite loads (as pEq./mL) with approach 1 (middle top), using interpolated parasite loads with approach 2 (middle bottom) and using interpolated values adjusted for sample volume, approach 3 (bottom). Right panels showing the relationship between Ct-values and parasite loads for a given interpolation approach for individuals with always detectable parasitemia (gray dots) and those alternating detectable/undetectable parasitemia (red dots).

Fig B. Sensitivity analyses for E1224-Placebo arm, D2

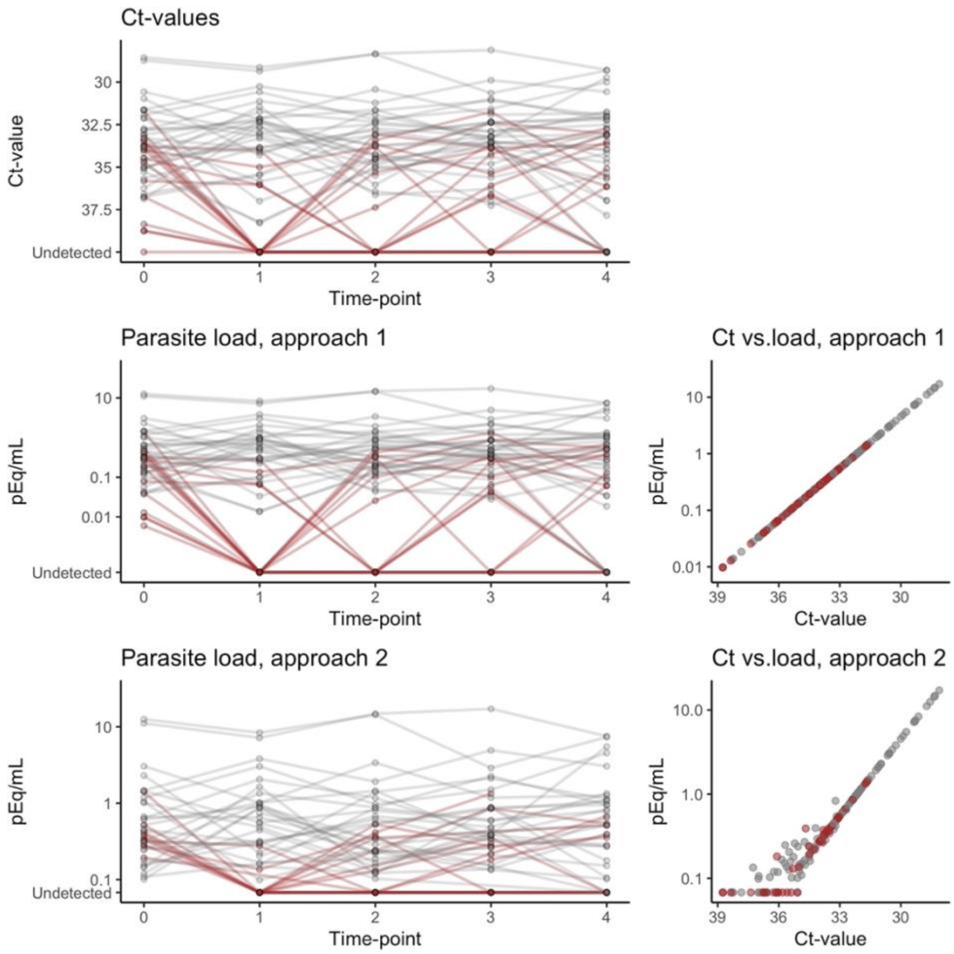

Fig B. Left row panels showing individual trajectories over time among those in D2 with always detectable parasitemia (gray lines) and those alternating detectable/undetectable parasitemia (red lines) when using Ct-values (top), using interpolated parasite loads (as pEq./mL) with approach 1 (middle) and using interpolated parasite loads with approach 2 (bottom). Right panels showing the relationship between Ct-values and parasite loads for a given interpolation approach for individuals with always detectable parasitemia (gray dots) and those alternating detectable/undetectable parasitemia (red dots). Log 10 scale is used for the y-axis to facilitate visualization.

Fig C. Distribution of Ct-values relative to sample volume in the Barcelona Cohort

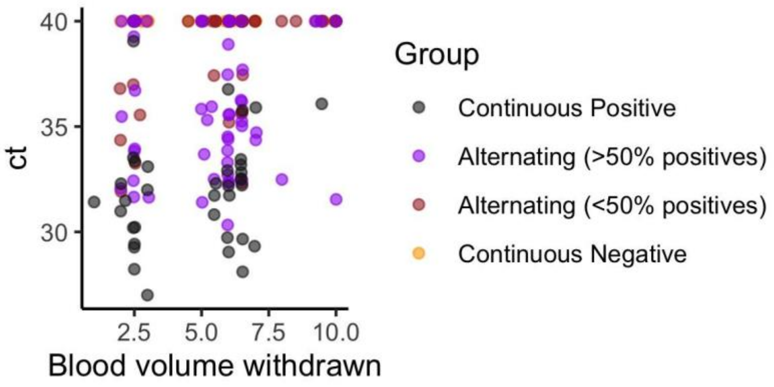

Supplement: S3 Text — Fig A in S3 Text. Sensitivity analyses for the Barcelona Cohort, D1. Left row panels showing individual trajectories over time among those in D1 with always detectable parasitemia (gray lines) and those alternating detectable/undetectable parasitemia (red lines) when using Ct-values (top), using interpolated parasite loads (as pEq./mL) with approach 1 (middle top), using interpolated parasite loads with approach 2 (middle bottom) and using interpolated values adjusted for sample volume, approach 3 (bottom). Right panels showing the relationship between Ct-values and parasite loads for a given interpolation approach for individuals with always detectable parasitemia (gray dots) and those alternating detectable/undetectable parasitemia (red dots). Fig B in S3 Text. Sensitivity analyses for the E1224-Placebo arm, D2. Left row panels showing individual trajectories over time among those in D2 with always detectable parasitemia (gray lines) and those alternating detectable/undetectable parasitemia (red lines) when using Ct-values (top), using interpolated parasite loads (as pEq./mL) with approach 1 (middle) and using interpolated parasite loads with approach 2 (bottom). Right panels showing the relationship between Ct-values and parasite loads for a given interpolation approach for individuals with always detectable parasitemia (gray dots) and those alternating detectable/undetectable parasitemia (red dots). Log 10 scale is used for the y-axis to facilitate visualization. Fig C in S3 Text. Distribution of Ct-values relative to sample volume in the Barcelona Cohort (PDF) [file pntd.0010828.s003.pdf]
