# Supplementary figures and images for: Human Trypanosoma cruzi chronic infection leads to individual level steady-state parasitemia: Implications for drug-trial optimization in Chagas disease
Source: PLoS Negl Trop Dis. 2022 Nov 21;16(11):e0010828. doi: 10.1371/journal.pntd.0010828 (PMC9721471; doi:10.1371/journal.pntd.0010828)

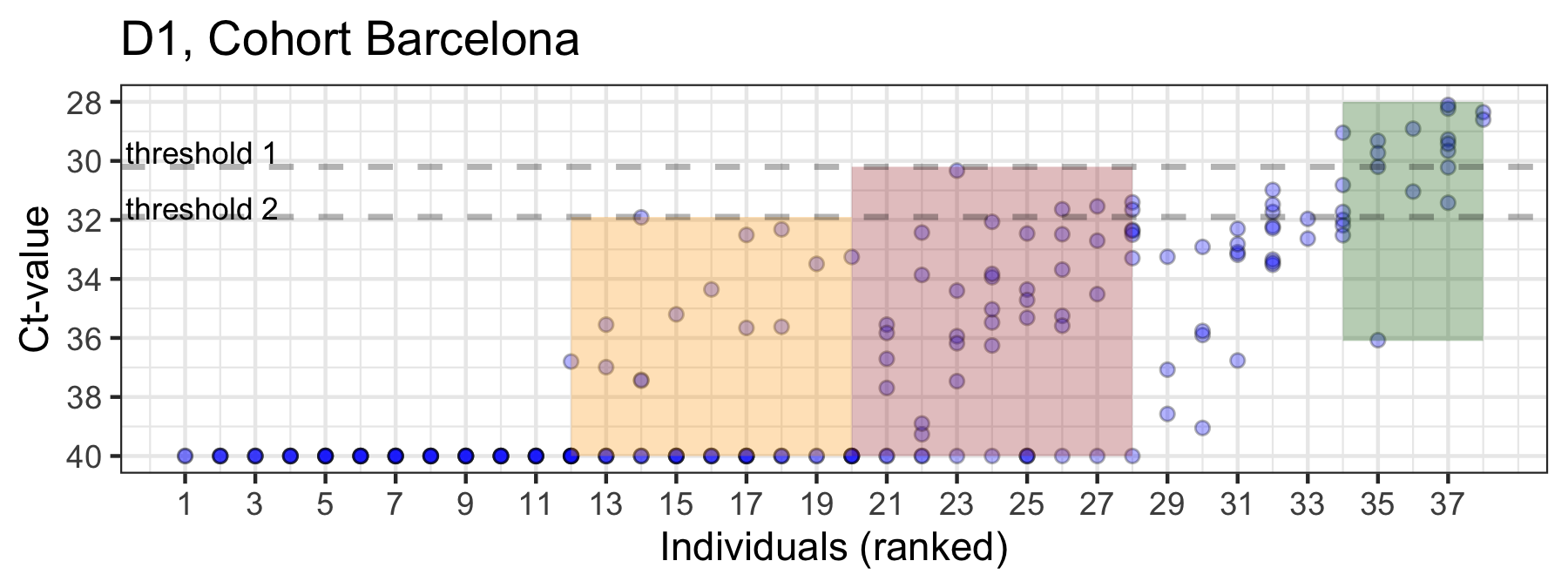

Supplement: S1 Fig — The red area represents individuals with alternating detectable/ undetectable parasitemia with >50% timepoints having detectable parasitemia, with the y-axis spanning 0 and the minimum Ct-value within the group. The green area represents individuals who always have detectable parasitemia, with the y axis showing the Ct-value range comprising that of individuals with continuous positive detection and at least one observation over the threshold 1. The orange area spans individuals with alternating detectable/ undetectable parasitemia with <50% timepoints having detectable parasitemia, and the y-axis spanning between 40 and the minimum Ct-value within the group (threshold 2). (TIF) [file pntd.0010828.s004.tif]
